# Transcriptional Analysis of Fracture Healing and the Induction of Embryonic Stem Cell–Related Genes

**DOI:** 10.1371/journal.pone.0005393

**Published:** 2009-05-05

**Authors:** Manish Bais, Jody McLean, Paola Sebastiani, Megan Young, Nathan Wigner, Temple Smith, Darrell N. Kotton, Thomas A. Einhorn, Louis C. Gerstenfeld

**Affiliations:** 1 Orthopaedic Research Laboratory, Boston University School of Medicine, Boston, Massachusetts, United States of America; 2 School of Public Health, Boston University Medical Center, Boston, Massachusetts, United States of America; 3 Department of Biomedical Engineering, Boston University School of Engineering, Boston, Massachusetts, United States of America; 4 Department of Medicine, Pulmonary Center Boston University School of Medicine, Boston, Massachusetts, United States of America; The University of Hong Kong, China

## Abstract

Fractures are among the most common human traumas. Fracture healing represents a unique temporarily definable post-natal process in which to study the complex interactions of multiple molecular events that regulate endochondral skeletal tissue formation. Because of the regenerative nature of fracture healing, it is hypothesized that large numbers of post-natal stem cells are recruited and contribute to formation of the multiple cell lineages that contribute to this process. Bayesian modeling was used to generate the temporal profiles of the transcriptome during fracture healing. The temporal relationships between ontologies that are associated with various biologic, metabolic, and regulatory pathways were identified and related to developmental processes associated with skeletogenesis, vasculogenesis, and neurogenesis. The complement of all the expressed BMPs, Wnts, FGFs, and their receptors were related to the subsets of transcription factors that were concurrently expressed during fracture healing. We further defined during fracture healing the temporal patterns of expression for 174 of the 193 genes known to be associated with human genetic skeletal disorders. In order to identify the common regulatory features that might be present in stem cells that are recruited during fracture healing to other types of stem cells, we queried the transcriptome of fracture healing against that seen in embryonic stem cells (ESCs) and mesenchymal stem cells (MSCs). Approximately 300 known genes that are preferentially expressed in ESCs and ∼350 of the known genes that are preferentially expressed in MSCs showed induction during fracture healing. Nanog, one of the central epigenetic regulators associated with ESC stem cell maintenance, was shown to be associated in multiple forms or bone repair as well as MSC differentiation. In summary, these data present the first temporal analysis of the transcriptome of an endochondral bone formation process that takes place during fracture healing. They show that neurogenesis as well as vasculogenesis are predominant components of skeletal tissue formation and suggest common pathways are shared between post-natal stem cells and those seen in ESCs.

## Introduction

Fractures are among the most common traumatic injuries in humans and osteoporosis related fractures are the fastest growing health care problem of aging [1]. The vast majority of fractures sustained worldwide are either untreated (e.g. patients in many third-world countries) or are treated in a manner that involves a periosteal response and a process of endochondral bone formation [2]–[4]. In contrast to most injury responses that lead to fibrotic scarring and incomplete restoration of tissue structure, fracture healing restores both structure and cell composition and in this regard is a true regenerative process. Over the time course of fracture healing, multiple cellular lineages that give rise to cartilage, bone, vascular, and hematopoietic tissues that make up a skeletal organ, are all recruited and contribute to the regeneration of the injured skeletal organ [5]–[9]. Because callus tissue formation is temporarily and spatially definable, fracture healing represents a unique process in which to study the complex interactions of the multiple molecular events that regulate endochondral bone formation.

Studies in different strains of mice have shown that there are genetically inherited variations in both the quantitative levels and the timing in expression of the transcription factors that control the differentiation of chondrogenic and osteogenic lineages during fracture healing process [10]. A large number of studies have shown that many of the individual morphogenetic factors and their receptors that control the embryonic development of skeletal tissues, such as Wnts, BMPs and FGFs, are also expressed during fracture healing [11]–[17] and that the manipulation of the expression of these morphogenetic factors will effect skeletal cell differentiation and the rate of fracture healing [11], [15]–[17]. The regenerative nature of fracture healing, the appearance of the primary morphogenetic factors that have been associated with embryological development of skeletal tissues, and the inherited nature in developmental variations of bone formation are all supportive of the generally held hypothesis that post natal fracture healing recapitulates the embryonic developmental processes that originally formed the injured skeletal tissues [5]–[7]. Consistent with this hypothesis is the general presumption that large numbers of stem cells are recruited and contribute to the development of the multiple cell lineages that contribute to bone healing.

A number of studies have exploited the unique spatial and temporal nature of callus formation to carry out large scale transcriptional profiling approaches to examine the expression characteristics of the transcriptome during the endochondral process of bone formation, which is observed during fracture healing. These previous studies however have shown two primary limitations: 1) either the number of time points that were used in their comparisons have been limited or; 2) very limited assessment approaches were used to relate the expressed genes to functional ontologies associated with specific cell lineages, biological processes, and molecular pathways [18]–[21]. As the use of transcriptional profiling has become more wide spread and as genomic and expression data bases have grown in scope, new technical approaches have been developed to more fully analyze and interpret large scale transcriptional profiling studies. The current study uses Bayesian modeling that clusters the expressed genes into groups that show temporally concurrent expression over multiple time points spanning a 21 day period of fracture healing. Multiple analyses were then carried out that defined the temporal dynamics of gene expression that were associated with various cell lineages, biological processes, regulatory and metabolic pathways. In this study, we also specifically define those Wnt, BMPs, and FGFs and their receptors that showed differential expression over the time course of fracture healing and related the expression of these genes to major groups of transcriptional regulators that were concurrently expressed with each of these families of morphogenetic genes. In order to determine if the patterns of expressed genes during fracture healing were reflective of stem cells, we compared the set of genes that were differentially expressed during fracture healing to the expression sets that are associated embryonic stem cell and mesenchymal stem cells. Finally, we place the expression of 174 of the 193 genes known to be associated with human genetic skeletal disorders in the context of their temporal expression during the process of endochondral bone formation that is seen during fracture repair.

## Materials and Methods

### Surgical Models

Research was conducted in conformity with all Federal and USDA guidelines, as well as an IACUC approved protocol. All studies were performed on male 8–10 weeks old C57 BL/6J (B6) mice. Age, sex, and genetic strain of mice were based on previous data, in which the time course of fracture healing had been established by both histological and selected candidate gene assessment [8]–[11]. Simple transverse closed unilateral fractures in the left femur of all mice were made using a modification of the method of Bonnarens and Einhorn [22], as previously described [10]. Surgical marrow ablation was carried out by reaming of the marrow space as described in Gerstenfeld et al., 2001 [23], using 15 week old male mice based on a modification of the method of Suva et al., 1993 [24], that had been developed for rats.

### MSC Culture and Osteoinduction

Marrow stromal cell cultures were prepared from C57 BL/6J (B6) male mice of 8–10 weeks of age (Jackson Laboratories, Bar Harbor, ME) and osteoinduction of these cultures as carried out as previously described [25].

### Murine Embryonic Stem Cell (ESC) Culture

Murine embryonic stem cells (E14.1; 129/Ola) [26–28) were maintained in the undifferentiated state by culturing in serum-free, feeder-free conditions as published [17]. This ESC line contains two knock-in reporter genes (Bry-GFP and Foxa2-hCD4) to allow monitoring of the differentiation state of cells in culture [26]. Briefly, the undifferentiated ESC were cultured on gelatin-coated 6-well plates in maintenance media^1^ consisting of 50% Neurobasal medium (Gibco-BRL) and 50% Dulbecco's Modified Eagle Medium/F12 medium (Gibco-BRL); with N2 and B27 supplements (Gibco-BRL), 1% penicillin/streptomycin, 0.05% bovine serum albumin, LIF (1000u; ESGRO; Chemicon), 10 ng/ml human BMP-4 (R&D Systems), and 1.5 10^−4^ M monothioglycerol (MTG) (Sigma).

### Murine Embryonic Fibroblast Cultures

Primary mouse embryonic fibroblasts (MEFs) were obtained using standard culture methods [29] for their isolation and expansion, from E13.5 mouse embryos (Charles River; strain #023; CF-1). Briefly, whole embryos were minced and trypsinized cells released from the tissue digestion were plated in fibroblast complete media (DMEM with 10% FBS; 1% penicillin/streptomycin and L-glutamine). Non-adherent cells were discarded, and plastic-adherent cells were serially passaged to purify and expand MEFs. All results shown are from passage 5 MEFs.

### RNA Preparation

After euthanasia, fracture sites were circumscribed by 5 mm on either side of the break. An identical region of tissue was excised from the mid half of the unfractured femora which was used as the control. Marrow ablation specimens were prepared by removing the distal cartilage condylar surfaces of the operated tibia and cutting approximately at the center of the mid-diaphyseal region. All bone tissues were collected into liquid nitrogen and stored at −80°C until used for RNA extraction. RNAs to be used for microarray were prepared as previously described [18]. RNA was prepared from cell cultures using the same basic procedure used for intact tissues except that at the time of harvest, the cell layers were directly scraped up into Trizol® (Invitrogen Carlsbad, CA) reagent and either directly extracted or frozen at −80°C

### Logistics of Transcriptional Profiling Analysis

**Figure 1:**
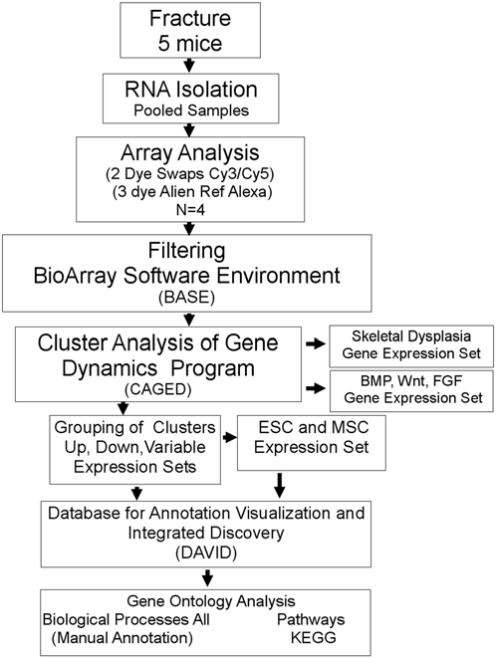
Flow chart of the logistics of the array analysis. Each of the major technical steps of the analysis is boxed. Arrows show the linear progression of the individual steps taken during the analysis. Programs used for each stage of the analysis are indicated in the parenthesis.

The basic technical approaches that were used in these studies are presented in Wang *et al*., 2006 [18]. A flow chart of the steps of this array analysis is presented in Figure 1. Large Scale transcriptional profiling was carried out on total RNAs that were collected across temporal profiles of fracture healing from C57 B6 mice. Specifically, we chose to study the expression profiles at day 0, 3, 5, 10, 14, and 21, where day 0 corresponds to unfractured bones and is used as the biological reference. For each time point, RNA from 5 mice was pooled and hybridized in quadruplicates, using a dye swap design to produce 4 expression measurements per time point.

### Microarray Hybridization and Image Analysis

Labeled samples were hybridized to slides containing the Operon Mus musculus probe set (Qiagen) of ∼21,000 70-mer oligonucleotides. All slides were co-printed with an internal “alien” sequence that has no sequence homologues in the mouse genome. Use of this alien allows an independent data set of absolute ratios of the individual hybridization intensities to be developed. Slide printing, array labeling, hybridization, and slide reading were performed at the Massachusetts General Hospital Genomics Core Facility as previous described [21]. All slides were quality control tested and contain appropriate positive and negative control sequences for data analysis. Ratio Intensities (RI) were directly determined by either using the cDNA produced from the unfractured bone as the biological reference or by using the externally added alien mRNA transcript as the reference. The alien gene in these studies serves as both a genome-extrinsic sequence and universal in-spot reference. In the experiments reported here, all microarrays were printed in which an alien 70 mer probe was co-printed with the different gene specific probes such that the alien was at a final concentration of 10% of the murine gene oligonucleotides. Hybridization was then carried out with cDNAs containing the complementary sequence of the alien oligo labeled with Alexa-488. This sequence was included into each of the two different nucleic acid test samples (RNA isolated from C57B6 mouse callus tissues and RNA isolated from C3H mouse callus tissues) that had been labeled with Cy3 and Cy5, respectively. There is in all four technical replicates with two dye swaps between the two strains of mice. Only data for the C57B6 strain is presented in this paper. All labeling was performed using a modified protocol of the Atlas Power script Fluorescent Labeling Kit (Clontech Cat # K1860-1). After labeling and slide hybridization, data was collected. A GenePix 4000B microarray scanner and software (Axon Instruments) were used to quantify micro arrays. Microarray data was stored and further quality controlled using the BioArray Software Environment (BASE). Raw and normalized data values are available through EBML-EBI repository, to be released in July 2009.

### Data Normalization and Filtering

Background was calculated locally per spot and subtracted from the intensity measurement of each hybridized spot. After background correction, negative expression values were removed and the data were normalized by scaling all individual intensities to a fixed target. Probes with less than 10 positive values after background correction were disregarded and the remaining expression data were normalized to the appropriate referent group. Final summaries per time point were computed as the average of the log10 normalized expression values.

### Post Array Data Analysis

The analysis of normalized values was carried out using the Cluster Analysis of Gene Expression Dynamics Program (CAGED, http://genomethods.org/caged/) [30]. The program uses a Bayesian models based clustering procedure to group genes into clusters, where genes assigned to the same cluster have the same temporal expression profile and therefore are likely to have similar functions, or common regulation. The clustering algorithm uses Bayes theorem to compute the probability that genes belong to the same cluster and details are described in Ramoni *et al*., 2002 [30]. A feature of the method is to use a cluster search procedure that automatically identifies the optimal number of clusters. We used the default setting of the program and filtered out all probes in which the temporal profile was always within 2 fold of expression from the referent group as our sample size does not allow reliable estimate of small effects [31]. We used polynomial models of degree 4 for modeling the temporal expression profiles that are better suited to describe short patterns [32]. Goodness of fit of the resulting model was assessed by checking the normality of the standardized residuals of each cluster.

**Figure 2:**
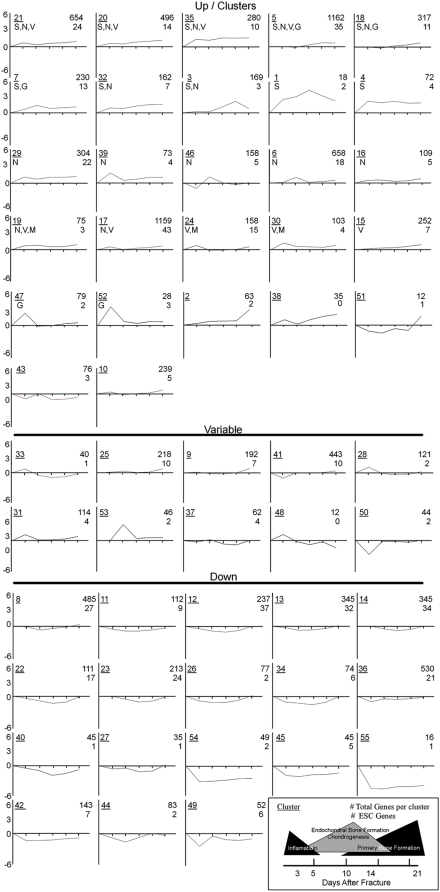
Temporal profiles of gene expression across fracture healing. Expression profiles of the temporal clusters produced by the Baysian modeling are presented. The three major groups of gene expression patterns (UP, Down and Variable) based on their temporal patterns of expression are presented. Biological ontologies associated with the development of specific tissue types (N = neurogenesis, V = vasculogenesis, S = Skeletogenesis, G = Gametogenesis) are indicated in the figure. All clusters in the variable group are specifically associated with T cell or B cell function or development. The underlined number is the cluster upper number is the total expressed genes in a cluster and lower number is the number of genes associated with ESCs. A diagrammatic presentation of the general biological stages of murine fracture healing, duration and relative scale of each stage are denoted in the figure in the lower right corner of the figure.

The clusters are summarized by the average expression profiles, per cluster, that is plotted against time (See Figure 2). This profile is computed as the average of expression, per time point, of all genes assigned to the same cluster.

**Figure 3:**
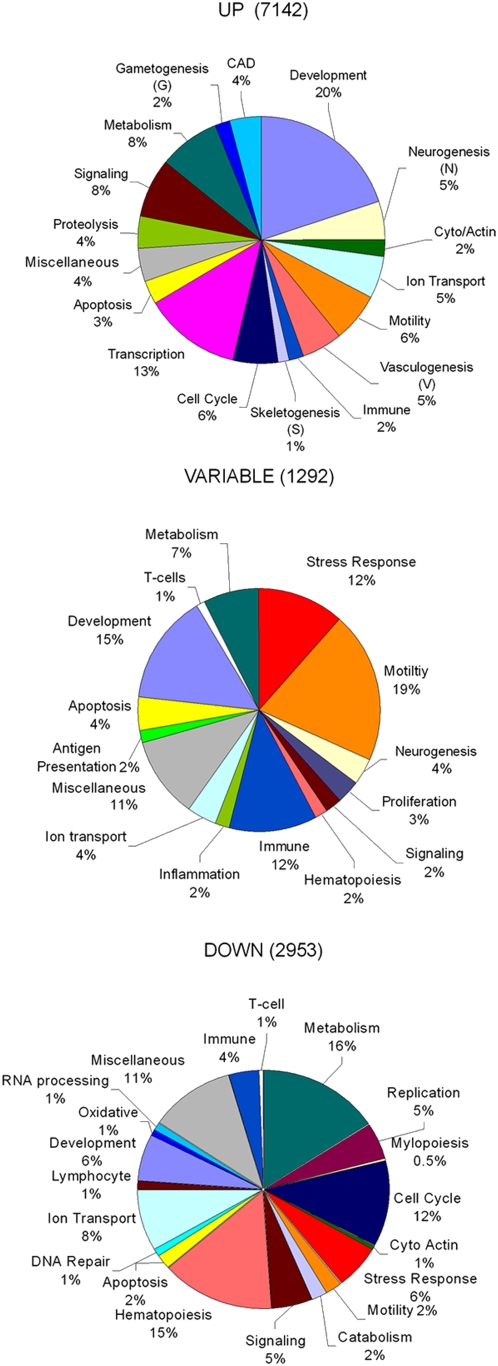
Distribution of biological functions associated with three major temporal grouping of gene expression. Pie Graph Distribution of biological functions was based on analysis of the three major groupings of the temporal clusters as indicated in Figure 1 using Database for Annotation, Visualization and Integrated Discovery (DAVID). Only those biological functional ontologies with p<.05 were considered with over lapping functions consolidated into single categories as indicated in Table S1. The total number of genes within each of the major groupings is indicated in the figure and percentage distributions of the grouping were calculated as descried in the materials and methods. A uniform color coding between the pie graphs for the biological groupings was used for comparisons between the groups.

**Figure 4:**
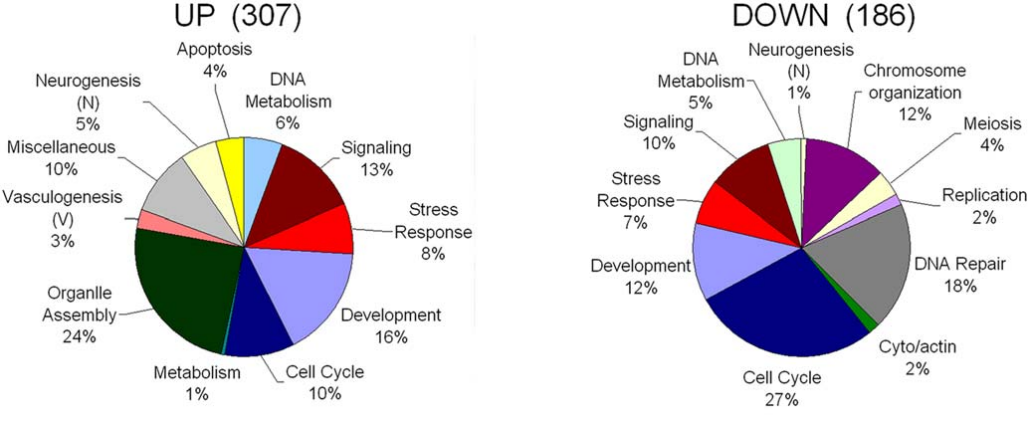
Distribution of biological functions associated with ESC genes identified as expressed during fracture repair. Two major temporal grouping of gene expression (Up or Down) is presented in a pie graph. Distribution of biological functions was based on analysis of the groupings of the temporal clusters as indicated in figure one using. Up and Variable groupings were consolidated together for analysis using Database for Annotation, Visualization and Integrated Discovery (DAVID). Only those biological functional ontologies with p<.05 were considered and those with over lapping functions were consolidated into single categories as indicated in Table S2. The total number of genes within each of the major groupings is indicated in the figure and percentage distributions of the grouping were calculated as descried in the materials and methods. A uniform color coding between the pie graphs for the biological groupings was used for comparisons between the groups.

The clusters of genes identified by CAGED were analyzed using the gene set enrichment analysis in David Bioinformatics resources Huang da *et al*., 2007 [33] (http://david.abcc.ncifcrf.gov/home.jspand), and Hosack *et al*., 2003 [34]. Only gene groups having an enrichment score ≤.05 were considered in this analysis. The ontologies with overlapping gene sets were manually annotated and grouped together into larger functional groupings (seen below in Figure 3 and Figure 4) that are summarized in Table S1 and Table S2.

### Candidate mRNA Expression Analysis

#### RT-PCR

All reagents for the PCR analysis were from Applied Biosystems, Inc., and plate assays were read in an ABI 7700 Sequence Detector (Applied Biosystems, Foster City, CA). The methods of DNA amplification were as previously described [18]. All samples were run in triplicates of pooled mRNAs from five mice for in vivo experiments. Cell culture measurements were from N = 6 derived from triplicates of two separate cell preparations. All reagents for the qRT-PCR analysis were from Applied Biosystems, Inc. Two μg of total RNA was used for each preparation of cDNA. All cDNA preparations were generated by random hexamer priming. Pertinent sequence information and amplicon sizes for all Taqman gene expression assays used in this study are available for each target gene from Applied Biosystems, Inc. (https://products.appliedbiosystems.com/ab/en/US/adirect/ab). Each plate contained two negative controls and a positive control probe. All mRNA levels were normalized to β-actin and each analysis was run in triplicate. The fractional cycle number at which the fluorescence passes the fixed threshold (C_T_ values) was used for quantification by using a comparative C_T_ method. This method is described within the Applied Biosystems, Inc. instruction manual for the instrumentation. Sample values are then normalized to the threshold value for βactin (Actb) for each time-point: ΔC_T_  =  C_T_ (exp)−C_T_ (Actb). The C_T_ value for day 0 was then used as a reference. ΔΔC_T_  =  ΔC_T_ (exp)−ΔC_T_ (exp day 0). The fold change in mRNA expression for each time point was plotted in a graph using day 0 as a reference: 2^−ΔΔCT (day 0)^  = 1.

## Results

### On Overview of the Temporal Dynamics of Transcriptional Activity during Fracture Healing

The set of data summarizing the total complement of expressed mRNAs in unfractured bone and within callus tissues at various times post fracture are summarized in Table S3. 11,432 mRNAs out of ∼18,346 expressed genes showed changed levels of expression across the time course of fracture healing at a non log transformed value of greater than +/−2 to unfractured bone. Based on these values and the current estimates of ∼38,000 genes in the mouse genome [35], these data suggest that a little over one half of the complement of genes expressed in the mouse genome were differentially regulated during fracture healing, while ∼80% of the mouse genome is expressed at a detectable level in skeletal organs. The Log ratio intensity values based on normalization to the in spike alien sequence control is also presented in Table S3. The externally normalized expression values to the co spotted alien sequence can be used to extrapolate copy number of expressed genes in the bone tissues. Approximately ∼10% of the gene descriptors in the array represent multiple splice variants that can also be deduced from the nucleotide sequences (Table S3). An example of the set of splice variants seen for the Runx2 is shown in the Figure S1. These results are used to provide a demonstration of how copy number values can be extrapolated from the externally normalized expression values.

The graphical appearance of the fifty five unique temporal clusters that were generated using the cluster analysis is seen in Figure 2 and a schematic of the general biological processes of murine fracture healing based on prior studies [8]–[11], [18], [36] is presented in the legend (bottom right). Twenty seven of these clusters showed patterns of increased expression across two or more time points and 18 clusters showed patterns of decreased expression across all time points. The remainder of the clusters were arranged in a variable group. Assignment to this group was based on two criteria. The first was that the cluster showed a pattern of increased or decreased expression at a single time point. The second criterion was that the expressed genes in the cluster showed a statistically relevant association with a biological ontology related to T cell function.

The pie graphs provide a global overview of the biological processes that were altered as a consequence of fracture (Figure 3). All biological ontologies that were identified and having a p≤.05 were individually examined and those that contained overlapping sets of genes and which were representative of redundant functions were grouped together for the simplified presentations of the pie graphs (see Materials and Methods and Table S1). The biological functions showing the largest percentage change across the whole time course of fracture healing were those associated with various developmental processes, specific elements of metabolism, and components of both intracellular and extracellular signaling. The three general groupings of the expression data showed that there were distinct differences that would define the cell or tissue types seen in each of these three broad groupings. Expression of genes associated with processes of vasculogenesis (V), neurogenesis (N), skeletogenesis (S), myogenesis (M), and gametogenesis (G) were all seen in the Up group of clusters. Gene expression associated with neurogenesis and vasculogenesis overlapped with the expressed groups of genes associated with all of these other tissue types, suggesting that the development of vascular and neural tissues were both concurrent and co-regulated with these other tissues. All of the clusters of the variable group had gene ontologies related to T-Cell functions. The variable group also contained some ontologies that were also associated with neurogenesis and vasculogenesis, but these are not denoted. The down clusters were selective for functional groups associated with myelopoiesis, specific aspects of T-cell function, B cell maturation and erythropoiesis.

Other processes, showing altered expression during fracture healing included the up regulation of gene families associated with cell adhesion (integrin based, gap junction based, and cadherin based) and actin cytoskeletal functions, while the variable group showed genes associated with motility related to expression of chemokimes, histocompatibility, innate immunity, antigen presentation, and stress response. The down regulated groups encompassed the biological processes associated with biotic defense responses, negative cell cycle regulators associated with DNA repair processes and erythropoiesis. Two other much more targeted types of analysis were carried out from these data. The first was to identify those ontologies associated with various metabolic functions that were distributed in the three temporal groupings of expression (Up, Down, or Variable). The list of these ontologies is seen in Table 1. The second analysis focused on the types of signal transduction or regulatory pathways that are associated with the expressed genes in the Up, Down, or Variable groups. This was carried out by comparing the gene expression data in the three groups to those pathway gene sets that have been defined in the Kyoto Encyclopedia of Genes and Genomes (KEGG) Pathways (http://www.genome.jp/kegg/pathway.html). The list of those pathways showing statistical significance in their expression across fracture healing is presented in Table 2.

**Table 1:** Variations in Metabolic Activities in the General Temporal Groupings

Up Groups	Variable Groups	Down Groups
Glucose transport	Arachidonic Acid (anabolic)	Porphyrin (anabolic)
Prostaglandin (anabolic)	Amino Acid Metabolism (modifying)	Pentose (catabolic)
Lipid (anabolic)	Amine Metabolism (anabolic)	Purine (catabolic)
Amino acid (anabolic)	RNA Metabolism (polymeric anabolic)	Pyrimidine (catabolic)
Nitric Oxide	Pyrimidine (catabolic)	Gluconeogenesis
Hexose transport		Alcohol catabolism
Catecholamine		Pyruvate metabolism (anabolic)
Dopamine		
Glucosamine		

**Table 2:** Identified KEGG* Pathways in the General Temporal Groupings

Up Groups	No. Genes	Variable Groups	No. Genes	Down Groups	No. Genes
ECM-receptor interaction	47	Antigen processing and presentation	15	Cell cycle (Negative Regulators)	35
Cytokine-cytokine receptor interaction	99	Arachidonic acid metabolism	9	Pentose phosphate pathway	12
Axon guidance	57	B cell receptor signaling pathway	8	Hematopoietic cell lineage	24
Melanogenesis	45	Cell adhesion molecules (CAMs)	16	B cell receptor signaling pathway	20
Focal adhesion	78	Cytokine-cytokine receptor interaction	27	Pyrimidine metabolism	23
TGF-beta signaling pathway	40	Glycine, serine and threonine metabolism	7	MAPK signaling pathway	51
Cell Communication	54	MAPK signaling pathway	23	Fc epsilon RI signaling pathway	20
Adherens junction	33	Natural killer cell mediated cytotoxicity	18	ABC transporters - General	13
Basal cell carcinoma	25	Pyrimidine metabolism	11	Leukocyte transendothelial migration	26
Complement and coagulation cascades	30	T cell receptor signaling pathway	12	Natural killer cell mediated cytotoxicity	27
Hematopoietic cell lineage	34	Type I diabetes mellitus	12	DNA polymerase	9
Glycan structures - degradation	14	VEGF signaling pathway	10	Melanoma	17
Olfactory transduction	14			Purine metabolism	27
GnRH signaling pathway	36			Porphyrin and chlorophyll metabolism	9
Renin-angiotensin system	10			Carbon fixation	7
Hedgehog signaling pathway	22			Focal adhesion	34
Sphingolipid metabolism	16			VEGF signaling pathway	15
Wnt signaling pathway	52				
Gap junction	34				

* Kyoto Encyclopedia of Genes and Genomes (KEGG) Pathways (http://www.genome.jp/kegg/pathway.html)

A large number of key regulatory mechanisms effecting skeletal tissue development have been identified over the last fifty years through the study of human congenital diseases [37]. Because many of these genes effect a wide variety of organ systems and do not have an overt relationship to skeletal tissue development, the analysis of their expression in fracture healing offers the unique possibility of identifying either the developmental stage at which the they effect skeletal tissues, or the potential co-interacting genes that provide complementation to the development of the skeletal phenotype. A basic assessment of all the currently known genes associated with human skeletal disorders was carried out to identify those known genes that are associated with congenital skeletal deficiencies that are differentially expressed during fracture healing. These data are summarized in Table S4 including the cluster that the gene is associated with and the major tissue ontologies associated with the cluster, and the type of phenotype that is displayed when the gene is mutated. Of the 193 genes that are currently classified as being associated with congenital disorder of skeletal tissues [37], 174 were shown to be present in the array profiles and 147 showed two fold or greater difference in expression during fracture healing. These data demonstrate that a number of types of disorders show associations with both unique temporal clusters and/or biological processes that are related with a given cluster. Thus, the genes associated with epiphyseal and metaphyseal dysplasias are almost all associated with clusters 1, 4, and 35 and these clusters are all associated with skeletogenesis and contain a preponderance of the expressed genes for extracellular matrix proteins. On the other hand, almost all of the genes that are associated with lysosomal disorders were associated with clusters 17 and 20 and these clusters were all associated with the development of multiple tissues.

### Temporal Relationships of the Expression of the BMP, Wnt, and FGF Morphogen Families to the Selective Expression of the Homeotic and Sox Transcription Factors during Fracture Healing

The expression patterns of the major morphogenetic families of proteins (BMPs, Wnts and FGFs) that are the known primary regulators of skeletal tissue development are next presented. This data is used as a means of validating that the temporal clustering accurately models the expression and interactions of specific genes, since the expression of many of these genes has been established from studies of embryological and post natal endochondral bone formation. The expression profiles of the BMP and Wnt gene families are first presented in Table 3. The individual genes for all of the expressed BMPs, Wnts, and their receptors are found in 11 clusters that are all in the unregulated group and are denoted in the Table 3. The expressed genes are arranged into four temporal sets: a set of from the clusters showing continuously unregulated expression over the time course of fracture healing (clusters 29, 32, and 35); a set from clusters showing a biphasic pattern that had a peak of expression at day 3 followed by a later increases at days 14 and 21 (clusters 17, 20;and 21); a set showing a middle pattern in which the genes had peak expression at 5 and 10 days (clusters 6 and 7); and a late set of genes showing peak expression from days 14 to 21(clusters 5, 15, and 18).

**Table 3:** TGFβ and Wnt Families of Expressed Ligands Receptors, Antagonists, Second Signals, and Transcription Factors

Temporal Pattern Clusters	Continuous			Biphasic			Middle			Late		
	29/32/35			17/20/21			6/7			5/15/18		
	BMP	Wnt	TF*	BMP	Wnt	TF	BMP	Wnt	TF	BMP	Wnt	TF
Receptors	Acvr1	Fzd6	Sox16	Acvr2	Fzd4	Sox4	Bmpr1a		Sox3		Fz8	Sox2
	Tgfbr2	Fzd2		Acvr2b	Fzd5	Sox7	Bmpr1b		*Sox5*			Sox5
				Tgfbr1	Fzd3	Sox10			Sox8			Sox9
				Tgfbr2		Sox12						Sox14
				Tgfbr3		Sox17						
				Bmpr2		Sox18						
Ligands	Tgfb3	Lrp1	*Prrx1*	Bmp4	Lrp12	Hoxa3			Pax1	Bmp15	Lrp12	Pax6
	Bmp1	Wnt5a	*Prrx2*	Bmp2	Lrp5	Hoxa4			Pax8	Bmp6	Wnt11	Pax7
		Wnt2	Hoxa4	Bmp5	Lrp6	Hoxa10			Pitx3	Gdf5	Wnt2b	Pitx2
			Hoxa11	Bmp8a	Lrp4	Hoxb1			Prrx1	Gdf7	Wnt7b	Hoxa1
			Hoxb2	Gdf9	Wnt5b	Hoxb13		Wnt9a	Prrx2		Wnt10b	Hoxa2
			Hoxb8	Gdf10	Wnt8b	Hoxc6					Wnt9b	Hoxa4
			Hoxc8			Hoxc10						Hoxa5
						Hoxd3						Hoxb3
Antagonists	Inhbb			Nog		Hoxd13	Inhba	Dkk3		Inhbe		Hoxb6
	Chrd		Sp7			Sp6	*Inhbb*	Wif	Rbp1			Hoxb9
	Bambi		Msx2			Twsg1			Ror1			Hoxc5
			*Dlx5*			Dlx5			Ror2			Hoxd3
			Mdfi			Dlx6						Hoxd9
			Nfatc1			*Mdfi*						
			*Runx2*			Nfatc2						
			Snai1			Ptch1						
Second	Smad1		Twist1	Smad3	Catna	Runx1				Smad4		
Signals			*Nf1*	Smad5	Catnb	Runx2				*Smad5*		
			Twist1	Smad6		Nf1				Smad7		
						Nf2						
			*Rora*			Rora	Rara					Rarg
						Thra						Tshr
						Sstr1						Sstr5
						Sstr2						
Other Descriptors	Rank	Apase	Rankl	Flt1	Jag	Ghr	Mint	Pou2f2		Calcb	Egf	Calcr
	Bsp	Igf1	Opn	Creb1	Igfr2	Pou6f1	EgfrlGf1			Vegfc	Crem	Shox2
	Jag2	Dmp	Csf1	Hif3a	*Crem*	Mmp9	Ctgf1			Creb3	Pou2f1	Sdfr2
	Col1a1	Nrp1	Col5a1	Dcn	Ngfr		PthrP			Pou5f1	Lmx1	
	Oscar	Bgn		Wisp1	Wisp2					Pparg		

Minor splice variant by quantitative expression are denoted by italicized text. * Transcription Factors

A number of examples for the expression of specific genes in these groups of clusters are presented to provide validation of the Bayesian modeling. The first example is related to the group of clusters that is expressed in the middle temporal period. The expression of Bmpr1a, Bmprb, PthrP, and Ctgf within this temporal grouping of clusters is concurrent with the initial proliferative phase of chondrocyte development during fracture healing and is consistent with both the known patterns of expression of these genes and their functional interactions during embryonic and growth plate endochondral bone formation [38]–[40]. A second example is related to the biphasic set of clusters. This set contained BMP 2, 4, 5, and 8a. We have previously shown that this particular group of BMPs is all co-expressed and regulated as a network during marrow stromal cell differentiation, and this network is under the control of BMP2 [16]. The multiple transcription factors that were identified in this set of clusters also have been shown to be reciprocally co-regulated by BMP2 and included Dlx5, Runx2, and Sp6 [41]–[44]. It is also of considerable interest that the major Wnt co-receptors Lrp5 and Lrp6 that are known to be associated with skeletal tissue development were also co-expressed with these BMPs and each other in these clusters, since a number of studies have shown the interaction of these families of morphogenetic proteins during osteogenesis as well as in embryogenesis [45]–[47]. A third example of the consistency of the temporal expression data to the published literature is related to the expression of BMP6 and Wnt 10b. These morphogens have been implicated in the regulatory control of osteoblast/adipocyte differentiation of the marrow stromal cells and are co-expressed during coupled remodeling [48]–[49]. The late appearance of their expression would be consistent with the initiation of coupled remodeling during the first wave of primary bone formation.

In the context of the expression of the BMP and Wnt morphogen families, these data also demonstrate that large numbers of homeotic transcriptional regulators and multiple members of the Sox family not previously shown to be post-natally expressed are seen during fracture healing. All of the Sox family members that were expressed, during fracture healing were isolated in the same clusters as the BMPs and Wnt families, while over 95% of the observed homeotic genes that showed altered expression during the time course of fracture healing were also in these clusters. In summary, the data presented in Table 3 provide a strong validation of the Bayesian modeling by demonstrating that the data had predictive value of many previously identified interactions of genes that had been examined in the context of their regulatory function in skeletal tissue formation.

The summary of the expression data for the FGF family of morphogenetic proteins and their receptors is presented in Tables S5. Several interesting observations may be made from these data. The first is that a large number of expressed FGF ligands are down regulated during fracture healing. This implies that these ligands must carry out functions in the normal bone tissue that are down regulated during the fracture healing process. The second observation is that all four FGF receptors and their splice variants are unregulated during fracture healing and their expression overlaps completely with the same clusters in which the BMPS and Wnts and their receptors are expressed. In contrast, while some of the FGF ligands are expressed in the same clusters as the Wnts and BMPs, a number of the FGF ligands are not. Finally, a large number of FGF isotypes that are differentially expressed during fracture healing are identified, which have not to date been characterized in the context of skeletal tissue formation.

### Multiple Genes Preferentially Expressed in ESCs Are Expressed During Fracture Healing

Since it may be speculated that large numbers of post natal stem cells are recruited during fracture healing, we sought to assess whether the transcriptional expression profiles seen during fracture healing would contain many of the genes that are preferentially expressed by stem cell populations. This question was first queried by carrying out a comparison of ∼12,000 genes showing a >2 fold change in the callus tissues to the set of genes that are preferentially expressed in embryonic stem cells (ESCs) [50]. This analysis identified that ∼300 out of ∼1000 genes known to be preferentially expressed in human embryonic stem cells (ESCs) were upregulated during fracture healing, while an additional ∼100 genes that showed preferential expression in ESC were downregulated. The distribution and numbers of these expressed genes within the different clusters of fracture healing are depicted in Figure 4. Table 4 presents a list of 17 genes common in ten or more transcriptional profiles of ESCs that are preferentially upregulated in fracture repair. We then compared two other transcriptional analysis studies that had defined those genes that showed preferential expression either in embryonic or post natal cell populations of MSCs [51], [52]. Table 5 identifies that subset of expressed genes common in fracture repair, ESCs, embryonic MSCs and post embryonic MSCs, and fracture healing. Only one expressed gene (methyl-CpG binding domain protein 2) overlaps between all four published expression profiles, while there was overlap between 12 expressed genes observed in ESC and embryonic MSCs. Table S6 presents the complete data sets comparing fracture callus mRNA expression to the three types of stem cells.

**Table 4:** Selective Set of Upregulated Genes in Fracture Healing Common to ESC

Pou5f1(Oct4)	POU domain, class 5, transcription factor 1	5
Dppa3	Developmental pluripotency associated 4	36
Sema6a	Semaphorin 6A	25
Gal	Galanin	36
Gabrb3	Gamma-aminobutyric acid (GABA) A receptor, beta 3	5
Sox2	SRY (sex determining region Y)-box 2	5
Lect1	Leukocyte cell derived chemotaxin 1	1
Nanog	Nanog Homeotic gene	43
Cyp26a1	Cytochrome P450, family 26, subfamily A, polypeptide 1	16
Zic3	Zic family member 3 (odd-paired homolog, Drosophila)	19
Pim2	Pim-2 oncogene	37,22
Aass	Aminoadipate-semialdehyde synthase	5
Usp9x	Ubiquitin specific peptidase 9, X-linked (fat facets-like, Drosophila)	5
Crmp1	Collapsin response mediator protein 1	17
Bmpr1a	Bone morphogenetic protein receptor, type IA	7
Orcil	Origin recognition complex, subunit 1-like (yeast)	43

Gene Symbol* Name Cluster

* Listed genes seen in only 10 or more transcriptional profiles of ESC cells and common to that subset of genes up regulated during fracture healing.

**Table 5:** Upregulated Genes in Fracture Healing Common to MSC and ESC+

Pgk1	phosphoglycerate kinase 1	5
Rplp0	ribosomal protein, large, P0	9
Enah	enabled homolog (Drosophila) (multiple splice variants)	17,21 35
Prdx1	peroxiredoxin 1	17
Uchl1	ubiquitin carboxy-terminal hydrolase L1	21
Tkt	transketolase	23
Lmnb2	lamin B2	27
Calu	calumenin (Calu), transcript variant 1	29
Cda	cytidine deaminase	29
Dpysl3	dihydropyrimidinase-like 3	29
Tpm1	tropomyosin 1, alpha	29
Mbd2#	methyl-CpG binding domain protein 2	13

Gene Symbol * Name Cluster

* Listed genes are common between that set of genes found in embryonic MSCs, ESC, and showing upregulated expression in fracture healing [51], [52]

# Common to ESC embryonic MSC and postnatal MSC

Figure 4 presents the graphic distribution of biological ontologies that are associated with the distribution of ∼300 upregulated and ∼100 down regulated genes that are expressed in the fracture callus tissue. The biological processes related to specific tissues that were seen in the UP group were those associated with neurogenesis and vasculogenesis. A very large group of genes were also associated with nucleoli and mitochondria organelle assembly and regulation of cell cycle control. Interestingly, a number of genes that are preferentially expressed in ESCs and which are seen in unfractured bone are downregulated after fracture. These genes are predominantly associated with negative regulatory mechanisms associated with cell cycle progression and DNA packaging. In the context of the nature of these gene's functions, it might be speculated that their downregulated expression from a basal level of expression in unfractured bone may be representative of the changes in expression of the quiescent population of stem cells as they are mobilized from their niche.

### Assessment of Two Candidate Stem Cell Associated Genes (Nanog and Methyl-CpG Binding Domain Protein 2) during in Skeletal Tissue Repair

**Figure 5:**
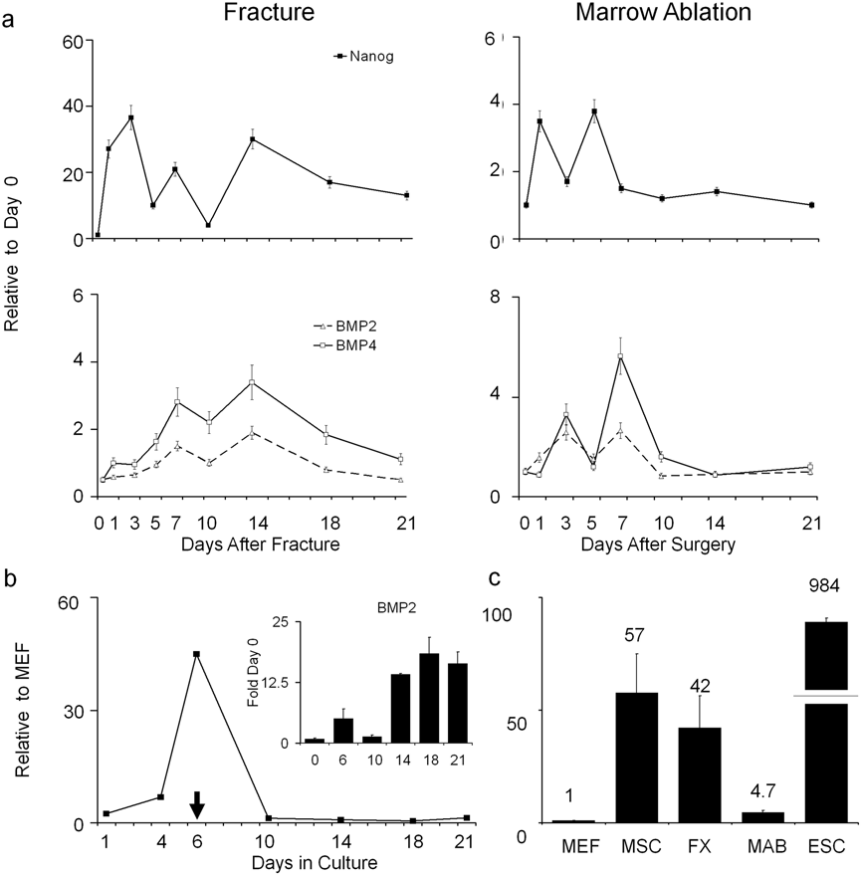
Comparison of steady-state Nanog mRNA expression relative to BMP2 and BMP4 mRNA expression levels over the time course of bone development in different models post-natal skeletal tissue formation. (A) Two in vivo models of injury induced bone formation. Fracture is presented on the left and marrow ablation is presented on the right. Time after fracture or surgical marrow ablation is indicated in the figure. Nanog profiles are in the top panel and BMP profiles are in the bottom panels. RNA measurements are presented as a relative fold of expression to day 0. Error bars  =  SD of three replicate measurements. (B) Relative mRNA expression of Nanog across the 21 time course of MSC differentiation. Arrow indicates time point at which media was switched to differentiation promoting media containing 10^−8 ^M Dexamethasome, 5mM β-GPO_4_, and ascorbate. Inset shows the comparisons of BMP2 levels across the same time of MSC differentiation. Levels of Nanog expression were made relative to murine embryonic fibroblasts while BMP levels were made in comparison to Day 0. c) Comparison of Nanog expression levels between various in vivo models of injury induced of post natal bone formation and in vitro models of MSC differentiation. All levels of expression were made at the time point at which maximal Nanog expression was observed. All levels are expressed relative to murine embryonic fibroblasts. Fold difference to MEFs are indicted by the numbers over each bar. Groups are MEF =  murine embryonic fibroblasts: Far left shows the levels of ESCs for comparison. Differences in ESC scale are denoted by broken bar. Mean fold levels different from MEFs are numerically denoted above each bar in the figure. Marrow Stromal Cells  = MSCs. Fracture callus tissues at 14 days post fracture  =  FX: Marrow ablation tissues at 5 days post surgery =  MAB. Error bars  =  SD of three replicate measurements.

The final set of studies were directed at validating the expression of two different candidate genes Nanog and Methyl-CpG binding domain protein 2. The data related to Nanog is first presented since it has been shown to be a central component in maintaining pluripotency of ESC and is related to repression of ESC response to BMP signaling [53]. The expression of Nanog was examined in both an endochondral model of bone formation (fracture healing) and a model predominated by intramembranous bone formation (marrow ablation). The expression of Nanog was compared to that of BMP2 and BMP4, since elevated BMP levels were shown to promote ESC differentiation [54], [55] (Figure 5). Both models showed multiple peaks of Nanog expression. Comparison of the fold differences in expression from their base lines showed that the post fracture levels of induction were almost ten fold greater than marrow ablation, with a ∼40 fold induction seen in fracture and maximal induction of ∼4 fold seen in marrow ablation. In both models of injury induced bone formation, each peak of Nanog expression was generally followed or concurrent with a peak of BMP2 and BMP4 expression.

Since the marrow ablation and fracture callus tissues from which the RNAs were purified contain complex mixtures of multiple cell types, it was unclear if Nanog expression would be representative of populations of cells that contribute to skeletal tissue formation or would be found in stem cells that give rise to other cell lineages. In order to further address this question, the expression of Nanog was examined over the time course of induction of osteogenesis of marrow stromal cells in vitro (Figure 5B). Messenger RNA levels are expressed relative to murine embryo fibroblasts (Mefs), considered to be a null or a typical differentiated cell that has minimal Nanog expression [56]. Nanog expression showed a ∼50 fold peak at Day 6, the time when the last of the non adherent cells were removed from the marrow cultures, but before they have been switched to their differentiation media. As soon as the osteogenous was induced and autogenously produced BMP2 levels rose, Nanog levels fell during the terminal period of MSC differentiation. Panel C presents a comparison of the peak levels of Nanog expression in MSCs, fracture callus tissues and marrow ablation tissues relative to those seen in Mefs or in comparison to ESCs. MSCs and fracture callus tissues had ∼50 fold greater expression than Mefs. While bone tissues from marrow ablation showed levels of induction only five fold those seen in the Mefs, the levels seen during skeletal tissue repair were many fold over Mefs but still about ∼20 fold less than ESCs.

**Figure 6:**
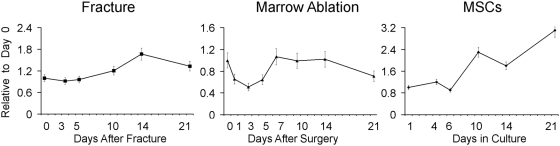
Comparison of steady-state mRNA expression levels of Methyl-CpG binding domain protein 2 over the time course of bone development in different models post-natal skeletal tissue formation. Two in vivo models of injury induced bone formation, Fracture (left panel) and marrow ablation (middle panel) are compared to the 21 time course of MSC differentiation (right panel). Time after fracture, surgical marrow ablation, or time after plating in culture are indicated in the figure RNA measurements are presented as a relative fold of expression to day 0. Error bars  =  SD of three replicate measurements.

The final data that is presented is for the Methyl-CpG binding domain protein 2 Gene expression (Figure 6). This gene was examined since it was common to both MSCs and ESCs in the three published studies to which we compared our expression data. In our cluster analysis study, this gene showed a downregulated expression over the time course until day 14, after which it returned to slightly above its base line expression in unfractured bone at day 21. When we looked at its expression by qRT-PCR within the two in vivo models of bone healing a very different pattern of expression from that seen for Nanog was observed. These results showed in both models that the expression of Methyl-CpG binding domain protein 2 rose at much later periods during bone healing than Nanog. Comparing the in vivo pattern of expression to that seen during in vitro MSC differentiation showed that its expression also increased late during the MSC culture's differentiation. This very different pattern of expression from Nanog suggests that it may not be expressed by the same cell populations as those expressing Nanog.

## Discussion

### An Overview of the Biological Processes of Response to Fracture

A number of previous studies have used transcriptional profiling approaches to identify novel candidate genes expressed during fracture healing [18], [57], [58]. This study however is the first to model the temporal profiles of gene expression and characterize the biological processes and pathways of the gene expression over the endochondral process of bone formation during fracture healing. Bayesian model based clustering of the transcriptome was used to establish the temporal relationships between the development of the multiple tissues that make up skeletal organs with the regulatory pathways and changes in tissue metabolism during fracture healing. The general metabolic picture of bone repair is one in which gluconeogenesis and nucleotide catabolism are down regulated while glucose and hexose transport, aerobic metabolism and the anabolic process involved in the synthesis of amino acids, lipids, proteoglycans and RNA are all increased. Several specialized metabolic processes show changed activities as an immediate response to the injury such as arachidonic, prostaglandin, and nitric oxide metabolism. On the other hand, catecholamine and dopamine metabolism most likely are related to aspects of neural tissue regulation and decreased porphyrin synthesis likely reflects diminished erythropoiesis.

The various tissue types, major biological processes (Figure 3), and various regulatory pathways (Table 1 and Table 2) that are related to fracture healing were broadly identified in this study. Although we do not provide an in-depth analysis of these data, an example of how this data might be used is related to understanding immune system response to injury. The fracture procedure used in these studies produced a robust transient inflammatory and innate immune response in an aseptic environment. In the context of this injury, activation of the acquired system does not take place. It is interesting to note however that groups of genes associated with B cell receptor signaling, natural killer cell mediated cytotoxicity, and T cell receptor signaling pathways are all transiently activated, while a second group of genes in these same pathways are all down regulated. Thus a comparison of the different sets of genes in these pathways that are either up or down regulated, provide insight into what gene expression is absent when acquired response activation does not take place versus when tissue repair takes place. In this context, recent studies have shown that bone marrow mesenchymal stem cells exert considerable immune regulatory activity [59] and in part are related to controlling autoimmunity. Finally these data may provide considerable insight into the common complication of immunosuppression that accompanies large trauma to the skeleton [60].

### The Role of Neurogenesis during Bone Formation

While it was anticipated that skeletogenic processes would predominate during fracture healing, the number of genes associated with vasculogenesis and neurogenesis associated processes were five times more prevalent than those associated with skeletogenesis. Genes associated with these biological processes also appeared to be expressed earlier during fracture healing than those associated with skeletogenesis and overlapped into the genes sets that were identified to be common with ESC. While it may be speculated that many of the developmental genes that control neurogenesis and vasculogenesis will overlap with the subset of genes associated with skeletogenesis, it is noteworthy that large numbers of the expressed genes seen in the neurogenic ontologies were unique structural elements associated with axon formation and neural signal transduction. Such data suggest then, that neurogenic tissues are also regenerated during fracture healing, which has considerable implications in terms of using this model to study specific aspects of post natal neural regeneration.

The early temporal expression of large numbers of genes associated with vascular and neurogenic tissue formation during fracture healing is consistent with the very early and concurrent development of these tissues during embryogenesis. In this context, salamander limb regeneration does not occur in the absence of accompanying neural tissue development [61], [62]. Individual genes that are upregulated during fracture healing that have been associated with both neural tissue development and limb development included N-Myc1 (neuronal myc) and the paired-like homeodomain transcription factors. In the case of N-Myc1, previous studies have associated this gene's expression within mammalian embryonic limb development [63], while the paired-like homeodomain transcription factors that are identified in our study have also been shown previously to be involved in amphibian limb regeneration [62]. As an aside, it is interesting to speculate that fracture healing is the phylogenetic descendant in mammals of the ontological processes of limb regeneration seen in amphibians.

Several studies have shown that bone and marrow tissues contain both myelinated and non-myelinated fibers that enter the bone along with blood vessels from the central feeding vessel along with many smaller branches from the periosteum that all terminate in the marrow space [64]–[66]. In a more recent study, the autonomic innervation of the marrow space was mapped back to the central nervous system [67] showing that there was central control of the nerve fibers that innervated the marrow. Nerve fibers containing neuropeptide Y2 [66] calcitonin gene related peptide, substance P, and dopamine [68], [69] have all been found in bone tissues, and in a recent studies either germ line or specific hypothalamic deletion of neuropeptide Y2 receptors has been shown to lead to greater trabecular bone volume [70]. Finally, multiple lines of evidence suggest that central neural control, integrally regulates aspects of bone marrow function and immunological response [71], [72] and a number of studies have established that there is a central control mechanism that regulates bone remodeling [73], [74].

### Commonality of Gene Expression between ESC and Post-Natal Stem Cells

Fracture healing is generally believed to recapitulate embryological mechanisms that formed the skeletal tissue. This raises the question whether the post natal stem cells that contribute to this repair process would express genes common with ESC or other types of stem cells. In this regard, it is noteworthy that almost a third of all genes that are preferentially expressed in ESCs are seen in fracture callus tissues and that three of these genes Oct4, Sox2, and Nanog, which play central regulatory roles in ESC maintenance [53] are observed to be up regulated during fracture healing. Two possibilities might explain the expression and functional role of these ESC genes in post natal animals. One possibility is that populations of stem cells persist from embryological development within the post natal bone and that these cells maintain a pattern of embryological gene expression that becomes apparent when large numbers stems cells are mobilized and expanded during skeletal repair. Alternatively, many of the genes that are expressed in ESC are induced by injury within specialized post natal cell populations that contribute to the regeneration of the skeletal tissues. While our observations do not demonstrate functionality of these genes in a post natal context, the examination of one of these genes Nanog was shown to be induced in different forms of injury induced skeletal repair and during marrow stromal cell differentiation in vitro. The biphasic manner in which Nanog was expressed was also consistent with the occurrence of separate waves of skeletal and hematopoietic stem cell differentiation that occur during both fracture healing and marrow ablation. Two recent studies suggest that there is a reciprocal relationship between Nanog and BMP's ability to promote differentiation. In one study, Nanog was shown to block BMP-induced mesoderm differentiation of ESC cells by interacting with Smad1 and interfering with the recruitment of co-activators to the active Smad transcriptional complexes thus preventing BMP induced mesodermal differentiation of ESCs [54]. In the second, more recent study the forced expression of Nanog in the C2 myogenic cell line did not alter terminal muscle differentiation but prevented the osteogenic differentiation of these cells when treated with BMP4 [75].

Lastly, we observed expression of large numbers of genes associated with gametogenesis and gonad function during fracture healing. While this observation is surprising, it is noteworthy that spermatogonia arise in the only known post natal stem cell niche that maintains a pluripotent stem cell population [53]. Thus our observation offers the intriguing possibility that some of the biological processes that maintain the spermatogonial stem cell niche may share common features with those found within skeletal tissues.

In summary, these data present the first temporal analysis of the transcriptome of an endochondral bone formation process that takes place during fracture healing. Major observations include: the extensive involvement of neurogenesis and vasculogenesis during injury induced bone repair; the overlap in genes that are upregulated to those that are preferentially expressed in ESC; and the characterization of the temporal expression patterns of genes known to be associated with various skeletal dysplasia during fracture healing.

## Supporting Information

Figure S1 Comparison of Expression Profiles of Runx2 By Two Methods of Normalization. An example of the set of splice variants seen for the Runx2 is shown in Figure S1. These results are used to provide a demonstration of how copy number values can be extrapolated from the externally normalized expression values. A comparison of the temporal expression of the four unique Runx2 transcripts based on the normalization of the expression data to either day 0 no fracture or the in spike alien control is presented. These data demonstrate the differences in the expression profile data based on the two different approaches of expression normalization. As can be seen from the data in which the values were externally normalized to the alien control, the two mRNA transcripts expressed in cluster twenty one were quantitatively the most prevalent. Based on the oligonucleotide sequences and their position in the genomic sequence four separate model sequences for the Runx2 mRNA were predicted. The primary splice differences in the exon arrangements for this mRNA were seen in variations in the mid to 3 prime regions of the mRNA. Interestingly the model prediction for the expressed transcript seen in clusters 21 and 35 were similar. In contrast the predicted model structure for the mRNA seen in cluster 32 showed an alternate initiation site for the coding sequence. Top panel are expression values based on normalization to the in spike alien oligonucleotide. Bottom panel are expression values based on normalization to day 0 no fracture. The legend denotes the expression profile lines for each of the four unique oligonucleotides used to assess splice variants for the Runx2 gene.
(0.04 MB PDF)

Table S1 Identified Biological Ontologies Used To Define the General Percentages of Functional Activities Across Fracture Healing. Table S1 presents the individual biological processes that were grouped together for presentation in Figure 3. The percentages for representative ontology groups that were identified using DAVID were determined for the total complement of expressed genes contained in the up, down and variable group of clusters (Table 4). Only those categories of biological processes with p≤.05 were considered and were sorted into categories representative of overlapping processes. The individual genes were identified for each biological process in a given group and a complete list of all genes was compiled for each category and all duplicates were removed. The percentage of each category was computed by the genes per category over the total number of genes for the three cluster subgroups included in Table 1 and Table 2. The first sheet are the percentage distributions of the consolidated biological groupings as well as separate break downs of the make up the signaling and metabolism groups into separate components that make up these grouping are presented. Table S1A Tabular percentages as assessed for the three groups (UP, Down, Varaible). Table S1B Identified Biological Groups Up. Table S1C Identified Biological Groups Down. Table S1D Identified Biological Groups Variable.
(0.08 MB XLS)

Table S2 Identified Biological Ontologies Used To Define the Percentages of Functional Activities Seen in Commonly Expressed Genes Between Fracture Healing and Those that Are Preferentially Expressed in ESCs. Table S2 presents the individual biological processes that were grouped together for presentation in Figure 4. The percentages for representative ontology groups that were identified using DAVID were determined for the total complement of expressed genes contained in the up, down and variable group of clusters (Table 4). Only those categories of biological processes with p≤.05 were considered and were sorted into categories representative of overlapping processes. The individual genes were identified for each biological process in a given group and a complete list of all genes was compiled for each category and all duplicates were removed. For this analysis the Variable and UP clusters were grouped together into a single category denoted as UP. The percentage of each category was computed by the genes per category over the total number of genes for the cluster subgroups. Table S2A: Identified Biological Groups Up and Variable (Consolidated into UP). Table S2B: Identified Biological Groups Down.
(0.08 MB XLS)

Table S3 Tabular Summary of the Expressed Genes Across the Time Course of Fracture Healing. Expressed gene sets are arranged by numeric cluster from the CAGED analysis. Unigene number, short letter gene name and gene name are denoted. Two sets of values are presented: Log ratio intensity values based on normalization to Day 0 no fracture: Log ratio intensity values based on normalization to the in spike alien sequence control. The sequence of each oligonucleotide is presented for assessments of nature of splice variants. These data are broken down to identify the subsets that was filtered and used for the clustering analysis. The externally normalized expression values to the co spotted alien sequence can be used to extrapolate copy number of expressed genes in the bone tissues. Approximately ∼10% of the gene descriptors in the array represent multiple splice variants that can be deduced from the nucleotide sequences (Table S3). The major biological themes that tracked with individual clusters may be identified by self examination of the clusters by assessments of the expressed genes in the cluster with DAVID. As one example, all of the major extracellular matrix proteins associated with cartilage tissue formation (col2a1, col9a1,2,3, col10a1, and aggrecan) tracked in cluster one. Table S3A: Tabular list of those genes that were spotted on the array which met inclusion criteria for use in the cluster analysis. Table S3B: Tabular list of gene expression data that did not meet criteria for inclusion in the cluster analysis but which were shown to have met the criteria for measurable expression. Table S3C: Tabular list of those genes that were spotted on the array but which did not meet inclusion criteria for measurable expression.
(8.49 MB XLS)

Table S4 Expression Characteristics of Known Genes Associated With Skeletal Disorders During Fracture Healing [37]. The list of skeletal disorders as summarized in Superti-Furga and Unger, 2007, was compared to those expressed genes seen in fracture healing. The observed list is arranged based on the classification scheme presented by Superti-Furga and Unger, 2007. The listing of the data was sorted based on association with: temporal cluster; Cluster group (Up, down or variable); Specific developmental ontologies (neurogenesis N, vasculogenesis V or skeletogenesis S; a simplified pathological classification: Brachydactylies BD; CF cranial facial; Dysostosis DP; epiphyseal or epiphyseal dysplasias EM; Generalized or multi-tissue dysplasias GDP; limb length differences LL; mineralization defects M; Polydactyly or Syndactyly; PS, synostoses SYN; or Vertebra or rib VR.
(0.13 MB XLS)

Table S5 Summary of the Expression of FGF Morphogenetic Family and Receptors Across the Time Course of Fracture Healing. The FGF morphogens and the FGF receptor are summarized based on clusters in which they are associated. Expressed genes are arranged by numeric cluster from the CAGED analysis. Unigene number, short letter gene name and gene name are denoted. Two sets of values are presented: Log ratio intensity values based on normalization to Day 0 no fracture: Log ratio intensity values based on normalization to the in spike alien sequence control. The sequence of each oligonucleotide is presented for assessments of nature of splice variants. Groupings of clusters are arranged by their temporal profiles in a similar manner as described for Table 3. Clusters showing continuous increases in gene expression are indicated by C: Clusters showing a biphasic pattern in gene expression are indicated by B: Clusters showing increased gene expression at middle time points 5 and 10 days post fracture are indicated by M: Clusters showing increased gene expression at late points 14 and 21 days post fracture are indicated by L. Clusters showing decreased gene expression throughout the post fracture period are indicated by D.
(0.03 MB XLS)

Table S6 Tabular Summaries of the Expressed Genes across the Time Course of Fracture Healing Showing Overlap With Those Preferentially Expressed in ESC, Embryonic MSCs or Post Natal MSCs. The overlap of those genes showing differential expression during the time course of fracture healing to three published gene sets of those showing preferential expression in ESCs and MSCs [35], [59], [60]. Expressed genes are arranged by numeric cluster from the CAGED analysis. Unigene number, short letter gene name and gene name are denoted. Two sets of values are presented: Log ratio intensity values based on normalization to Day 0 no fracture: Log ratio intensity values based on normalization to the in spike alien sequence control. The sequence of each oligonucleotide is presented for assessments of nature of splice variants. ESC: Expressed genes overlapping between those preferential to ESC and showing altered expression in fracture healing. MSC: Expressed genes overlapping between those preferential to post natal MSCs and showing altered expression in fracture healing. EMSC: Expressed genes overlapping between those preferential to embryonic MSCs and showing altered expression in fracture healing.
(0.35 MB XLS)
