# Supplementary figures and images for: Transcriptional Analysis of Fracture Healing and the Induction of Embryonic Stem Cell–Related Genes
Source: PLoS One. 2009 May 5;4(5):e5393. doi: 10.1371/journal.pone.0005393 (PMC2673045; doi:10.1371/journal.pone.0005393)

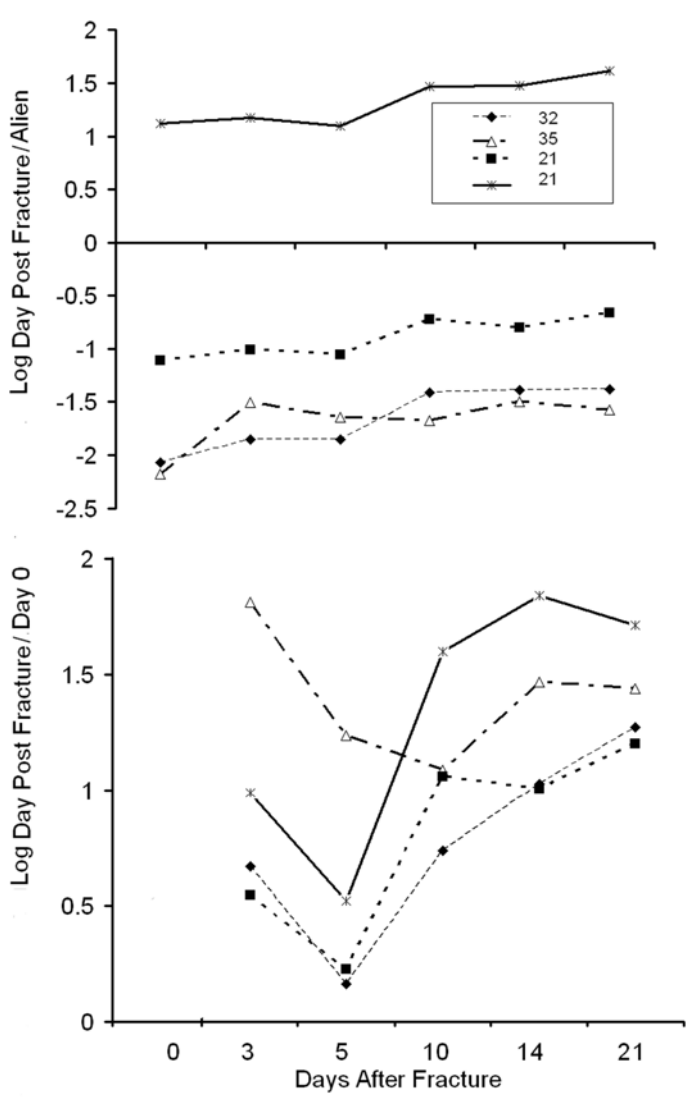

Supplement: Figure S1 — Comparison of Expression Profiles of Runx2 By Two Methods of Normalization. An example of the set of splice variants seen for the Runx2 is shown in Figure S1. These results are used to provide a demonstration of how copy number values can be extrapolated from the externally normalized expression values. A comparison of the temporal expression of the four unique Runx2 transcripts based on the normalization of the expression data to either day 0 no fracture or the in spike alien control is presented. These data demonstrate the differences in the expression profile data based on the two different approaches of expression normalization. As can be seen from the data in which the values were externally normalized to the alien control, the two mRNA transcripts expressed in cluster twenty one were quantitatively the most prevalent. Based on the oligonucleotide sequences and their position in the genomic sequence four separate model sequences for the Runx2 mRNA were predicted. The primary splice differences in the exon arrangements for this mRNA were seen in variations in the mid to 3 prime regions of the mRNA. Interestingly the model prediction for the expressed transcript seen in clusters 21 and 35 were similar. In contrast the predicted model structure for the mRNA seen in cluster 32 showed an alternate initiation site for the coding sequence. Top panel are expression values based on normalization to the in spike alien oligonucleotide. Bottom panel are expression values based on normalization to day 0 no fracture. The legend denotes the expression profile lines for each of the four unique oligonucleotides used to assess splice variants for the Runx2 gene. (0.04 MB PDF) [file pone.0005393.s001.pdf]
